# Assessment of cardiometabolic risk using single point insulin sensitivity estimator (SPISE) in pediatric Bardet–Biedl Syndrome: a pilot study

**DOI:** 10.1186/s13023-026-04353-y

**Published:** 2026-08-06

**Authors:** Tugce Kandemir, Melek Yildiz, Ummahan Tercan, Ozge Bayrak Demirel, Hasan Yanik, Volkan Karaman, Ayca Dilruba Aslanger, Aslı Derya Kardelen, Sukran Poyrazoglu, Feyza Darendeliler, Guven Toksoy, Firdevs Bas

**Affiliations:** 1 Department of Pediatric Endocrinology, Istanbul Faculty of Medicine, Istanbul University, Istanbul, Türkiye; 2 Department of Medical Genetics, Istanbul Faculty of Medicine, Istanbul University, Istanbul, Türkiye; 3 Department of Genetics, Institute of Graduate Studies in Health Sciences, Istanbul University, Istanbul, Türkiye

**Keywords:** Bardet–Biedl syndrome, Pediatric obesity, Syndromic obesity, Metabolic syndrome, MetS z-score, SPISE, Insulin resistance, Insulin sensitivity

## Abstract

**Background:**

Bardet-Biedl syndrome (BBS) carries early cardiometabolic risk, yet pediatric screening is complicated by growth and puberty. The metabolic syndrome (MetS) z-score provides a continuous benchmark for clustered risk. The single-point insulin sensitivity estimator (SPISE), based on body mass index (BMI) and fasting lipids, may offer a practical alternative where insulin testing is impractical. We aimed to assess the association between SPISE and cardiometabolic burden in children with molecularly confirmed BBS, and to compare its ability to identify MetS against the MetS z-score benchmark and insulin-derived indices.

**Methods:**

Single-center retrospective pilot study including children/adolescents with genetically confirmed BBS who underwent standardized anthropometry and metabolic profiling (fasting lipids, glucose, insulin; OGTT when available). SPISE–MetS z-score associations were examined using Spearman and adjusted analyses. In adolescents (≥ 10 years), MetS discrimination was evaluated with ROC curves for SPISE, TG/HDL, and homeostatic model assessment for insulin resistance (HOMA-IR), with pairwise DeLong comparisons and Youden-optimal thresholds.

**Results:**

Fourteen participants (7 females, 7 males) were evaluated. Median age at last visit was 11.7 years [IQR 7.6–15.5]; BMI SDS was 3.05 [2.47–3.57]. The median MetS z-score was 1.60 [0.90–1.75]. SPISE correlated inversely with the MetS z-score (ρ=-0.57, *p* = 0.021), and adjusted models (age, sex, BMI-SDS) retained significance. Among adolescents (*n* = 10), SPISE showed the highest AUC for MetS (AUC 0.95; 95% CI 0.82–1.00) versus TG/HDL (AUC 0.81) and HOMA-IR (AUC 0.60); pairwise differences were not statistically significant. Youden-optimal SPISE ≤ 3.34 identified MetS with high sensitivity in adolescents. Confidence intervals were wide, reflecting the small sample size.

**Conclusions:**

In this single-center pediatric BBS cohort, SPISE tracked continuous MetS burden and showed numerically stronger discrimination for MetS than insulin-derived indices. These findings highlight the potential utility of SPISE as a feasible tool for cardiometabolic monitoring in syndromic obesity, where laboratory access may be limited. Beyond BBS, SPISE may support early risk stratification and follow-up in rare obesity models of metabolic risk, but multicenter prospective validation remains warranted.

**Supplementary Information:**

The online version contains supplementary material available at 10.1186/s13023-026-04353-y.

## Background

Bardet–Biedl syndrome (BBS) is a rare autosomal recessive ciliopathy characterized by multisystem involvement, including retinal dystrophy, polydactyly, renal anomalies, hypogonadism, and early-onset obesity [1, 2]. Beyond these classic features, metabolic complications such as insulin resistance, dyslipidemia, hypertension, and type 2 diabetes emerge early in life and represent major contributors to long-term morbidity [3, 4].

Recent pediatric BBS cohorts report metabolic syndrome (MetS) in roughly half of patients, underscoring substantial early cardiometabolic risk [4]. The diagnosis of metabolic syndrome (MetS) in childhood remains challenging because conventional cut-offs differ across definitions and are strongly influenced by growth and pubertal development [5, 6]. To overcome these limitations, continuous severity scores such as the MetS z-score have been proposed, providing a more sensitive approach for quantifying clustered cardiometabolic risk [7].

In pediatric populations, insulin sensitivity is usually estimated through surrogate indices, including homeostatic model assessment of insulin resistance (HOMA-IR), the Matsuda index, quantitative insulin sensitivity check index (QUICKI), and Stumvoll equations. [8–13]. While informative, these measures require fasting blood tests and, in some cases, oral glucose tolerance testing, which can be burdensome in routine clinical care. The Single-Point Insulin Sensitivity Estimator (SPISE)—a lipid- and BMI-based index—was recently validated in adolescents and adults, demonstrating strong associations with insulin resistance and future diabetes risk in both general and obese populations [18, 19]. However, its utility in monogenic or syndromic obesity, such as BBS, has not been explored.

Here, we investigated the performance of SPISE in a genetically confirmed cohort of pediatric BBS patients. We specifically compared its ability to capture adverse metabolic status with established insulin-derived indices and the MetS z-score. We hypothesized that SPISE would correlate with MetS z-scores and demonstrate superior discriminatory capacity compared with HOMA-IR and Matsuda in identifying elevated cardiometabolic risk in this rare disorder.

## Methods

### Study design and setting

This retrospective single-center study was conducted in the Department of Pediatric Endocrinology, Istanbul University, Istanbul Faculty of Medicine. Patients followed from January 1987 to December 2024 were identified from institutional medical records.

### Participants and eligibility

**Fig. 1:**
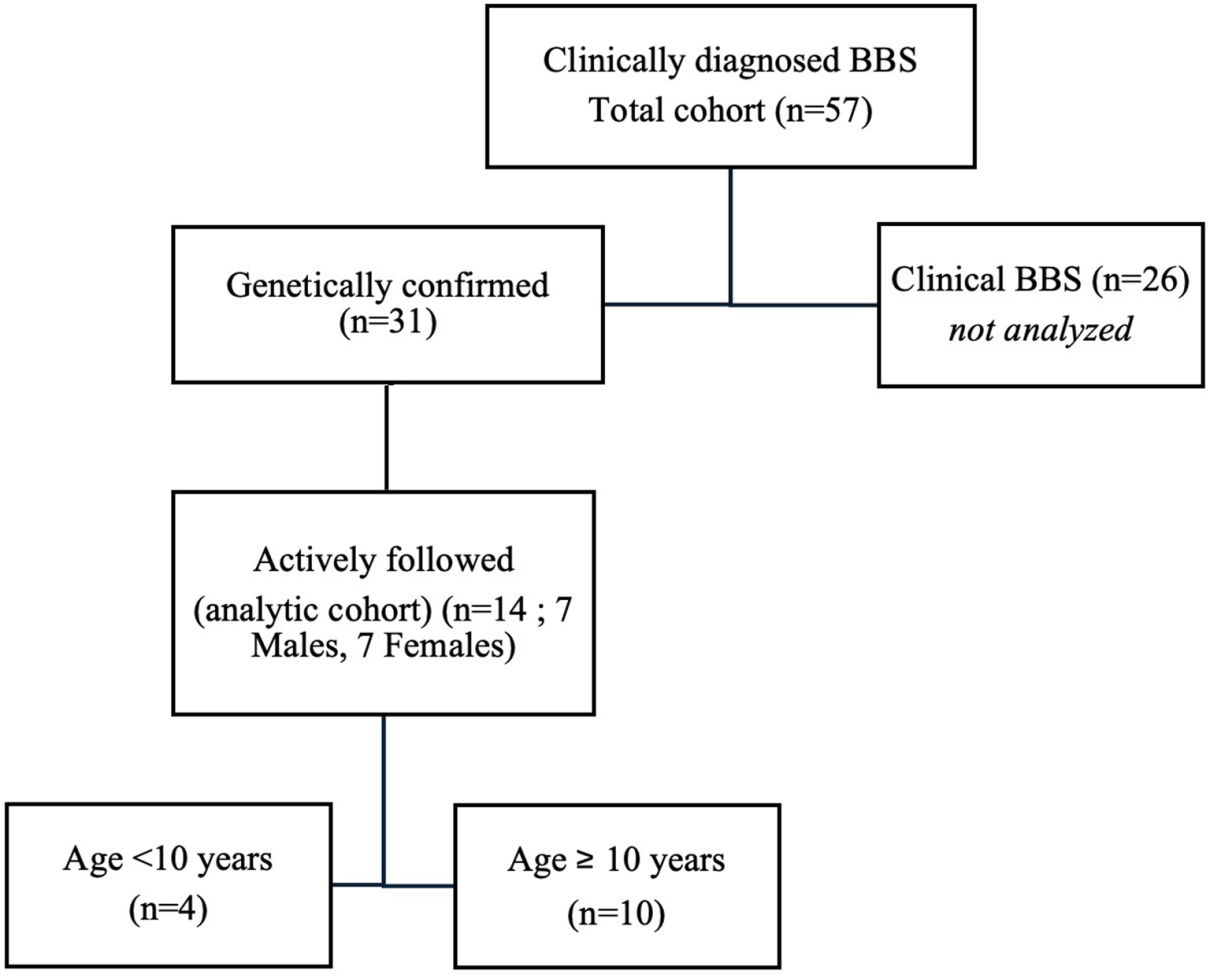
Cohort assembly and age- stratified analytic sets. legend: Of 57 patients with clinically diagnosed BBS, 14 had molecular confirmation and were under active follow-up; these constituted the analytic cohort. Because pediatric metabolic-syndrome definitions apply from ≥ 10 years, MetS-based analyses were restricted to participants aged ≥ 10 years (*n* = 10). Children < 10 years (*n* = 4) contributed descriptive summaries

Fifty-seven children and adolescents with a clinical and/or molecular diagnosis of BBS were screened. Of these, 31 had molecular confirmation, while 26 were diagnosed clinically. Inclusion criteria for the analytic cohort were: (i) molecularly confirmed BBS (biallelic pathogenic/likely pathogenic variants in a BBS gene according to American College of Medical Genetics and Genomics (ACMG) criteria) and (ii) active follow-up at our clinic, defined as ≥ 2 outpatient clinic visit within the previous 12 months with available anthropometry and fasting biochemistry. Exclusion criteria included loss to follow-up; uncertain molecular findings or isolated carrier status and missing core data. None of the participants were receiving antihyperglycemic, antihypertensive, or lipid-lowering pharmacotherapy, nor had any of them undergone bariatric surgery or received obesity-targeted treatments (including GLP-1 receptor agonists or setmelanotide) at the time of metabolic assessment. Fourteen patients (7 females, 7 males) from 12 unrelated families met the criteria and were included in the final analyses. Cohort assembly and the age-stratified analytic sets are shown in Fig 1.

### Age stratification and analytic sets

Because pediatric MetS definitions are recommended from age ≥ 10 years, MetS-based analyses were restricted to participants aged ≥ 10 years (*n* = 10). Children < 10 years (*n* = 4) were included for descriptive summaries and continuous risk metrics (e.g., MetS z-score) only and were excluded from categorical MetS outcomes.

### Metabolic evaluation

Fasting blood samples were obtained after an overnight fast (≥ 8 h) for glucose, insulin, HbA1C, lipid profile included low-density lipoprotein cholesterol (LDL-C), high-density lipoprotein cholesterol (HDL-C), total cholesterol, and triglycerides (TG) and liver enzymes. Blood pressure was measured in accordance with pediatric guidelines. When clinically indicated, an oral glucose tolerance test (OGTT; with 1.75 g/kg, maximum 75 g oral glucose solution) was performed with samples obtained at 0, 30, 60, 90, and 120 min [10]. Dyslipidemia was defined according to IDF pediatric criteria as fasting triglycerides ≥ 150 mg/dL and/or HDL-cholesterol < 40 mg/dL for children aged 6–15 years and for males aged ≥ 16 years, or < 50 mg/dL for females aged ≥ 16 years, per IDF adult criteria applied at that threshold [6]. Hepatic steatosis was diagnosed and graded by ultrasonography: grade 1 (mild), grade 2 (moderate), and grade 3 (severe), consistent with established sonographic criteria [7]. Hypertension (HT) was defined as systolic or diastolic BP ≥ 95th percentile according to the recent sex, age and height Table [8]. For adolescents ≥ 13 years and adults, HT was defined as systolic and/or diastolic BP ≥ 140/90 mmHg and elevated BP was defined as systolic and/or diastolic BP ≥ 120/80 mmHg according to 2017 guidelines [8, 9].

Cardiometabolic burden was summarized with the sex- and age-specific MetS z-score described by Gurka et al. [10, 11]. The score integrates waist circumference, TG, HDL-C, systolic blood pressure, and fasting glucose into a unitless continuous variable (mean = 0, SD = 1); thus higher values indicate greater clustered risk. We analysed the MetS z-score as a continuous measure; when a binary label was required for sensitivity analyses, a cohort-specific cut-point was derived by ROC (Youden). MetS z-score was analyzed as a continuous variable; for descriptive purposes, ‘elevated’ was defined as z ≥ 1.0 (= 84th percentile), recognizing that pediatric cut-points are not standardized.

### Metabolic indices

Insulin resistance and sensitivity were assessed using established surrogate indices. Given the absence of a universal pediatric cut-off and the influence of puberty, insulin resistance was modelled as a continuous variable. Calculations (glucose in mmol/L, insulin in µU/mL unless otherwise stated) included:

- HOMA-IR [12] = (fasting insulin × fasting glucose) / 22.5.
- HOMA-B [13] = (20 × fasting insulin) / (fasting glucose − 63).
- QUICKI [14] = 1 / [log(fasting insulin) + log(fasting glucose)]
- Matsuda index [15] = 10,000 / √[(fasting glucose × fasting insulin) × (mean OGTT glucose × mean OGTT insulin)]
- Insulin-to-glucose ratio (I/G) = fasting insulin / fasting glucose.
- Stumvoll β-cell function equations [16]:
  - First phase: 1283 + (1.829 × Ins_30) − (138.7 × Glu_30) + (3.772 × Ins_0).
  - Second phase: 287 + (0.416 × Ins_30) − (26.07 × Glu_30) + (0.922 × Ins_0).
- ISI (OGTT) index [17] = [1.9/6 × weight (kg) / fasting plasma glucose] + 520 − [1.9/18 × weight (kg) / 120-min glucose]
- IRI [18]: (fasting insulin x fasting glucose)/25.
- SPISE index [19, 20] = (600 × HDL^0.185^) / (TG^0.2^ × BMI^1.338^), with HDL and TG in mg/dL and BMI in kg/m².

### Genetic analysis

Molecular diagnosis was established by next-generation sequencing (NGS) panels targeting obesity- and ciliopathy-related genes. Eight patients were diagnosed through the Rare Obesity Advanced Diagnosis (ROAD) program. ROAD is a genetic diagnosis support program provided by Rhythm Pharmaceuticals for rare MC4R pathway diseases. Genetic testing was performed by Unilabs/CGC Genetics, an ISO15189 certified clinical laboratory. All variants were confirmed by Sanger sequencing and classified according to ACMG guidelines (as listed in Table 1).

**Table 1:** Genetic classification and identified variants in patients with Bardet–Biedl syndrome (n=14)

BBS subtype/Location	Nucleotide change(NM)	Peptide change(NP)	Zygosity	Loc.	ACMG Classification(Franklin)	RefSNPs (rs)	Ref.
BBS1 (n=1)11q13.1	NM_024649.5c.48-1G>A	NP_078925.3p.(?)	Homozygous	Exon 1	Pathogenic(PM3, PVS1, PM2)	rs751753112	Harville (2010) [21]
BBS2 (n=4)16q21	NM_031885.5c.1770_1791delins15	NP_114091.4p.(Phe590Leufs*29)ᵃ	Homozygous	Exon 14	Likely pathogenic(PVS1, PM2)	Novel	This study
	c.940del(Two cases)	p.(Ile314Serfs*11)	Homozygous	Exon 8	Pathogenic(PVS1, PM2, PP5)	rs58777824	Nishimura (2001) [22]
	c.1864C>T	p.(Arg622*)	Homozygous	Exon 15	Pathogenic(PM3, PVS1, PM2)	rs201196733	Deveault (2011) [23]
BBS4 (n=1)15q22.3-q23	NM_033028.5c.332+2_332+3insTT	NP_149017.2p.(?)	Homozygous	Exon 5	Likely pathogenic(PM3, PM2)	rs753360929	Knopp (2015) [24]
BBS5 (n=2)2q31.1	NM_152384.3c.974A>G(Two cases)	NP_689597.1p.(Glu325Gly)	Homozygous	Exon 12	Likely pathogenic(PP3, PM2)	Novel	This study*
BBS7 (n=1)4q27	NM_176824.3c.849+1G>T	NP_789794.1p.(?)	Homozygous	Intron 8	Likely pathogenic(PVS1, PM2)	-	Gumus (2021) [25]
BBS8 (TTC8) (n=1)14q31.3	NM_198309.3c.221_222dup	NP_938051.1p.(Ala75Serfs*16)	Homozygous	Exon 2	Likely pathogenic(PVS1, PM2)	Novel	This study
BBS9 (n=3)7p14	NM_198428.3c.310del(Three cases)	NP_940820.1p.(Cys104Valfs*20)^b^	Homozygous	Exon 4	Pathogenic(PVS1, PM2, PP5)	rs777498165	Kleinendorst (2018) [26]
BBS10 (n=1)12q21.2	NM_024685.4c.530A>G	NP_078961.3p.(Tyr177Cys)ᵃ	Homozygous	Exon 2	Likely pathogenic(PM3, PM2, PM1, PP3)	rs1555202700	Harville (2010) [21]

^a^ PCSK1 p.(Asn221Asp) heterozygous co-occurrence. ^b^ PCSK1 p.(Asn221Asp) heterozygous identified in 2 of the 3 cases.

*Segregation analysis showed heterozygous carrier status in both parents, consistent with the inheritance pattern.

Abbrev.: ACMG: The American College of Medical Genetics and Genomics, BBS: Bardet Biedl Syndrome, Loc: Location, Ref: Refference, —, not reported/not applicable. NM: RefSeq messenger RNA transcript, NP: RefSeq protein sequence, Variant nomenclature follows HGVS.

### Statistical analysis

Analyses were performed using IBM SPSS Statistics, version 30 (IBM Corp., Armonk, NY, USA). Continuous variables are expressed as medians with interquartile ranges, and categorical variables as numbers and percentages. Group comparisons were performed with the Mann–Whitney U test (continuous) or Fisher’s exact test (categorical). Correlations with MetS z-scores were assessed using Spearman’s rho.

Discriminatory performance of SPISE, HOMA-IR, and Matsuda index for adverse metabolic status (defined as MetS in participants ≥ 10 years or elevated MetS z-scores in the full cohort) was evaluated with receiver-operating characteristic (ROC) analysis. Area under the curve (AUC) with 95% confidence intervals was calculated, and pairwise comparisons were performed using the DeLong method. Youden’s J statistic was used to identify optimal cut-off points, with sensitivity, specificity, likelihood ratios, and predictive values estimated assuming an observed prevalence of 70%.

To further explore determinants of cardiometabolic burden, linear regression models were constructed with MetS z-scores as the dependent variable and age, sex, pubertal status, BMI-SDS, and SPISE as covariates. Logistic regression was additionally performed as a sensitivity analysis for the outcome of MetS in participants ≥ 10 years. Two-sided p-values < 0.05 were considered statistically significant.

## Results

### Cohort characteristics

Fourteen children and adolescents with molecularly confirmed BBS were included (7 females, 7 males). The median age at diagnosis was 4.9 years [IQR 2.3–15.5], and the median age at last visit was 11.7 years [7.6–15.5] after a follow-up of 5.3 years [1.4–9.8]. Parental consanguinity was reported in 78.5% of cases. The most frequent genotypes were BBS2 (28.6%) and BBS9 (21.4%) (Table 1).

### Anthropometry and metabolic status

At last evaluation, median BMI SDS was 3.05 [2.47–3.57], and waist circumference SDS was 4.00 [3.34–4.47]. Metabolic abnormalities were highly prevalent: dyslipidemia in 12/14 (85.7%), insulin resistance in 10/14 (71.4%), hypertension in 9/14 (64.3%), hepatosteatosis (4 patients grade 2; 2 patients grade 2–3; 1 patient grade 3 hepatosteatosis) in 7/14 (50.0%), and type 2 diabetes in 1/14 (7.1%) (Table 2). The median MetS z-score was 1.60 [0.90–1.75]. Among participants ≥ 10 years (*n* = 10), 7 (70%) fulfilled criteria for metabolic syndrome, while 2/4 (50%) of those < 10 years had elevated MetS z-scores. Detailed anthropometric and metabolic findings at the last follow-up visit are summarized in Table 3. Detailed clinical, anthropometric, hormonal, and metabolic characteristics of patients showed in Supplementary Table S1.

**Table 2:** Clinical and demographic profile of patients with Bardet–Biedl syndrome at the time of diagnosis

Characteristic	Value
Age, years	4.93[2.30-15.52]
Gender (n)	F: M:7/7
Consanguinity	11(78.5%)
Birth weight SDS	0.17[-0.45- 0.84]
Age at onset of weight gain, years	0.5[0.5–8.5]
BMI SDS at presentation	2.69[2.62–4.10]
Age at onset of puberty	9.26[7.9-11.93]
Duration of follow-up, years	5.31[1.44–9.83]
***Additional features (n out of 14 patient)***	
Brachydactyly	*4*
Syndactyly	*11*
Renal anomaly^a^	*10*
Chronic renal failure^b^	*4*
Psychomotor retardation	*12*
Epilepsy	*3*
Ataxia	*2*
Anosmia, hyposmia	*2*
Scoliosis	*3*

^a^Renal anomalies included: hypoplastic kidney (*n* = 1), calyceal dilatation/hydronephrosis (*n* = 5), horseshoe kidney (*n* = 1), fetal lobulation (*n* = 1), uterovesical fistulae (*n* = 1), cortical cyst (*n* = 1).^b^ Chronic kidney disease was defined as eGFR < 60 ml/min/1.73 m² on ≥ 2 measurements at least 3 months apart, consistent with CKD stages 3–5 (KDIGO criteria); two patients required renal transplantation. Data are presented as median [IQR] or *n* (%), unless otherwise stated. BMI: Body mass index, F:M, female-to-male ratio, SDS: Standard deviation score

**Table 3:** Clinical, anthropometric, and metabolic characteristics of patients at their last follow-up visit

Anthropometric features	Median [IQR]
Age, years	11.72 [7.55-15.52]
Weight SDS	2.74 [0.8-5.3]
Height SDS	0.19 [-5-1.6]
BMI SDS	3.05 [2.47-3.57]
Waist circumference (cm)	99 [81.7-115.6]
Waist Circumference SDS	4 [3.34- 4.47]
Pubertal status (n,%)	Prepubertal n=4 (28.5%), Pubertal n=10 (71.5 %)
Systolic BP, mmHg *	114.8 ± 12.7
Systolic BP SDS	0.81 [0.04- 1.88]
Diastolic BP, mmHg*	74.9 ± 8.8
Diastolic BP SDS	1.41 [0.51-1.84]
Endocrine comorbidities	n (%)
High blood pressure/hypertension	9 (64.3%)
Dyslipidemia	12 (85.7%)
Insulin resistance	10 (71.4%)
Type 2 diabetes mellitus	1 (7.1%)
Hepatic steatosis	7 (50%)
Subclinical/central/primary hypothyroidism	6 (42.8%)
Metabolic Syndrome	n (%)/ Median [IQR]
MetS (≥10 years old, n=10)	7 (70%)
MetS z-score	1.6 [0.9-1.75]
Elevated MetS z-score** (<10 years, n=6)	3 (50%)

* Variables with normal distribution were expressed as mean ± standard deviation, ** Elevated MetS z-score: z ≥ 1.0 (unitless; mean=0, SD=1; higher = greater clustered cardiometabolic risk), BMI: Body mass index, BP: Blood pressure, MetS: Metabolic syndrome.

### Correlations with MetS burden

**Fig. 2:**
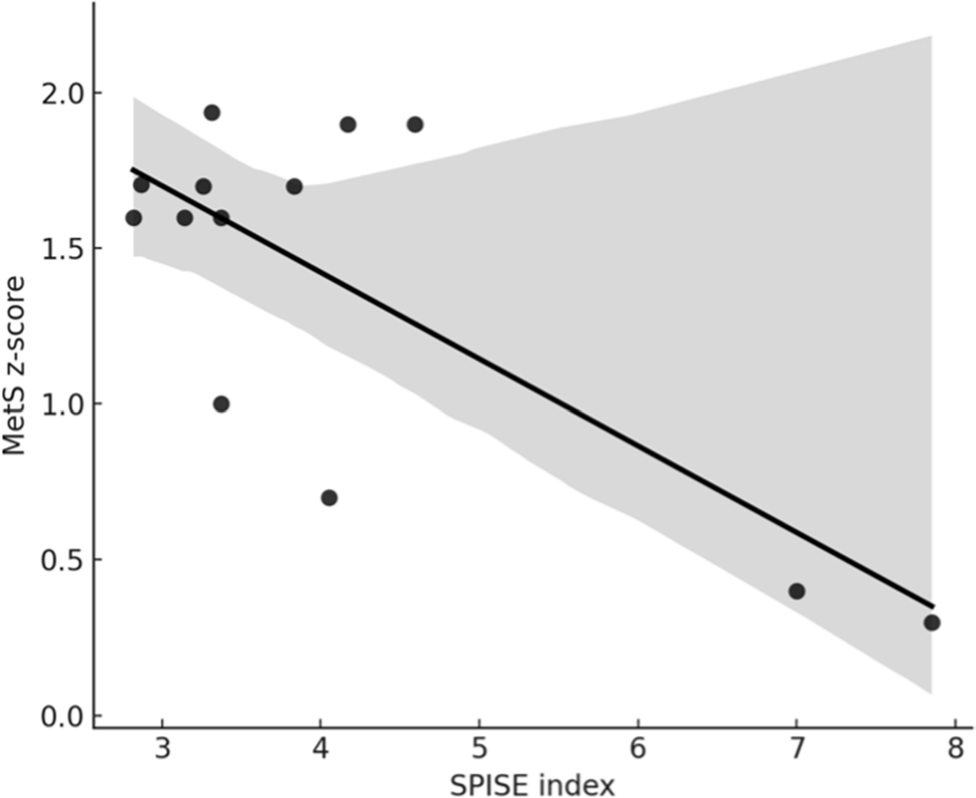
Relationship between SPISE index and MetS z-score. Legend: illustrates the negative correlation between SPISE and MetS z-scores.*Abbreviations: SPISE: Single-Point Insulin Sensitivity Estimator*,* MetS: Metabolic syndrome*

Across the whole cohort, higher SPISE was associated with lower MetS z-scores and lipid-based markers (MetS z: *r* = − 0.57, *p* = 0.021; TG/HDL: *r* = − 0.62, *p* = 0.017; TG/glucose: *r* = − 0.62, *p* = 0.018). The association with HbA1c was borderline (*r* = 0.48, *p* = 0.082). No significant associations were seen with insulin-derived indices (HOMA-IR, Matsuda, QUICKI, ISI, Stumvoll; all *p* > 0.30). Glucose metabolism parameters, including fasting glucose, insulin, and surrogate insulin sensitivity/resistance indices, are presented in Table 4. In the ≥ 10-year subgroup (*n* = 10), the direction of the SPISE–MetS z-score relation was similar but not significant (ρ = −0.563, *p* = 0.146; 95% CI − 0.912 to 0.259), reflecting limited precision due to the small sample. Figure 2 illustrates this negative relationship.

**Table 4:** Glucose metabolism parameters of patients’ last clinic visit (*n* = 11)

Parameters	Median/Mean ± SD*	IQR (25–75 percentiles)
HbA1c, %	5.5	5.27–5.6
Glucose, mg/dl	73.6 ± 7.7	
Insulin, µU/ml	48.7 ± 61.1	
**Indices**		
HOMA-IR	3.45	2.13–8.46
OGTT 120 min HOMA-IR	51.5	28.9–84.9
HOMA-B	1255	753–2193
HOMA-S	22.5	9.5–42.5
ISI	1.83	1.07–2.52
IRI	3.25	2.96–3.62
I/GI	1.31	0.91–1.99
QUICKI index	0.31	0.28–0.34
MCRest Stumvoll index	0.01	-0.04- 0.07
Matsuda index	1.74	1.03–2.29

* Variables with normal distribution were expressed as mean ± standard deviation, G= Glucose; I = insulin; HOMA-IR = homeostasis model assessment-insulin resistance; ISI = insulin sensitivity index (ISI composite); IRI = insulin resistance index; HOMA B = homeostasis model assessment-insulin secretion of the β-cell; 0, 30, 60, 90, 120 min = minutes from the start of the oral glucose tolerance test

### SPISE versus insulin-based references

When insulin resistance was defined by HOMA-IR, SPISE showed limited discriminatory ability (AUC 0.55, 95% CI 0.23–0.87; *p* = 0.751; Youden cut-off 3.84 → sensitivity 50%, specificity 75%). In contrast, using the Matsuda index as reference, SPISE yielded an AUC of 1.00 (*p* < 0.001), although this likely reflects sampling variability in the very small subset.

### Discrimination of metabolic syndrome (≥ 10 years)

Among participants aged ≥ 10 years, SPISE demonstrated stronger discrimination for metabolic syndrome than TG/HDL or HOMA-IR (AUCs: SPISE 0.95 [0.82–1.00]; TG/HDL 0.81 [0.41–1.00]; HOMA-IR 0.60 [0.09–1.00]). Pairwise DeLong comparisons were not statistically significant (SPISE vs. TG/HDL *p* = 0.532; SPISE vs. HOMA-IR *p* = 0.108; TG/HDL vs. HOMA-IR *p* = 0.545). Youden-optimal thresholds were SPISE ≤ 3.34, TG/HDL ≥ 5.54, and HOMA-IR ≥ 3.59 (see Supplementary Table S2). Because MetS definitions include TG, HDL, and fasting glucose, partial overlap with SPISE and HOMA-IR may have inflated AUCs, warranting cautious interpretation.

### Group comparisons and regression models

**Fig. 3:**
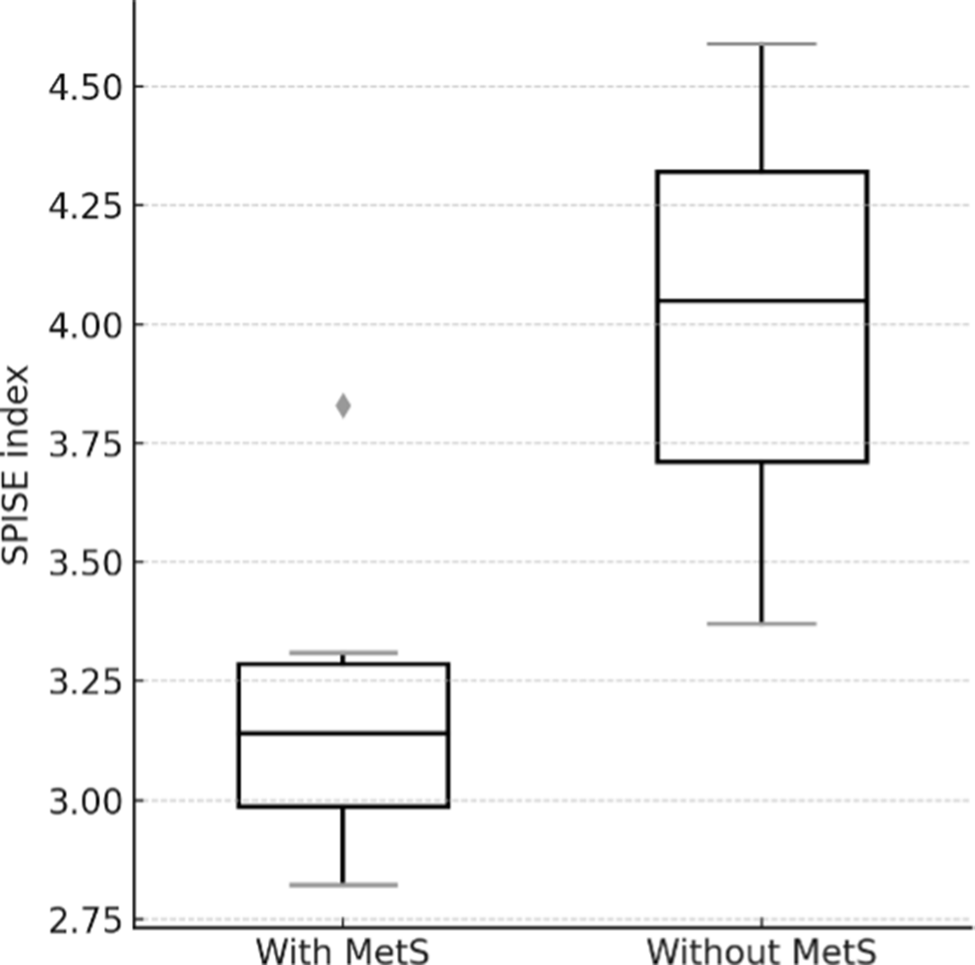
Compares SPISE values between patients with and without metabolic syndrome, demonstrating lower values in the affected group

SPISE values were significantly lower in children with metabolic syndrome compared to those without (median [IQR] 3.14 [2.90–3.40] vs. 4.05 [3.40–4.60]; *p* = 0.033; Fig 3). In linear regression, SPISE independently predicted MetS z-scores (β=−0.811, *p* < 0.001; R²=0.657). However, logistic regression in the ≥ 10-year age group yielded perfect classification (Nagelkerke R²=1.0), suggesting model overfitting and requiring cautious interpretation.

## Discussion

This is the first study to evaluate both the SPISE index and the MetS z-score in a genetically confirmed pediatric cohort with BBS, providing novel insight into early cardiometabolic risk in syndromic obesity. In this pediatric BBS cohort, cardiometabolic risk was evident from early childhood. Most adolescents met MetS criteria, and several younger children already showed elevated MetS z-scores. These findings support structured surveillance beginning in childhood and align with recent reports that rapid adiposity gain and dyslipidemia drive long-term morbidity in BBS [27]. This pilot, hypothesis-generating study in a rare-disease context aims to indicate the direction of effects rather than provide precise estimates. We used a pediatric MetS definition appropriate to our setting; because criteria differ across studies, cross-cohort comparisons are not straightforward, and a continuous severity approach helps bridge that heterogeneity [28]. The genetic architecture of this cohort deserves comment. All 14 patients harboured homozygous pathogenic variants, a pattern reflecting the high parental consanguinity rate (78.5%) characteristic of the Turkish population and consistent with prior reports from Turkiye [25]. The predominant BBS subtypes were BBS2 (28.6%) and BBS9 (21.4%), which contrasts with larger European and North American registries where BBS1, BBS7, and BBS10 are most frequent and compound heterozygous variants constitute a substantial proportion [4, 22, 23, 29]. Whether BBS subtype or zygosity modifies the metabolic phenotype and by extension, the performance of cardiometabolic indices such as SPISE is unknown. The homozygous BBS2/BBS9 predominance may limit generalizability to populations with heterozygous or BBS1/10-predominant genotype distributions, and genotype-stratified metabolic analyses in sufficiently powered cohorts remain warranted.

SPISE reflected the overall cardiometabolic risk pattern in a manner consistent with clinical experience. It was negatively correlated with the MetS z-score and to TG-based dysmetabolism (for example, TG/HDL), while its association with HbA1c was only borderline. In BBS, TG-high/HDL-low profiles commonly precede sustained hyperglycemia, so a stronger lipid signal than glycemic signal at this stage is plausible [4]. The adolescent subgroup showed a similar trend, but the confidence intervals were wider, consistent with limited precision due to small sample size rather than a biological difference.

**Fig. 4:**
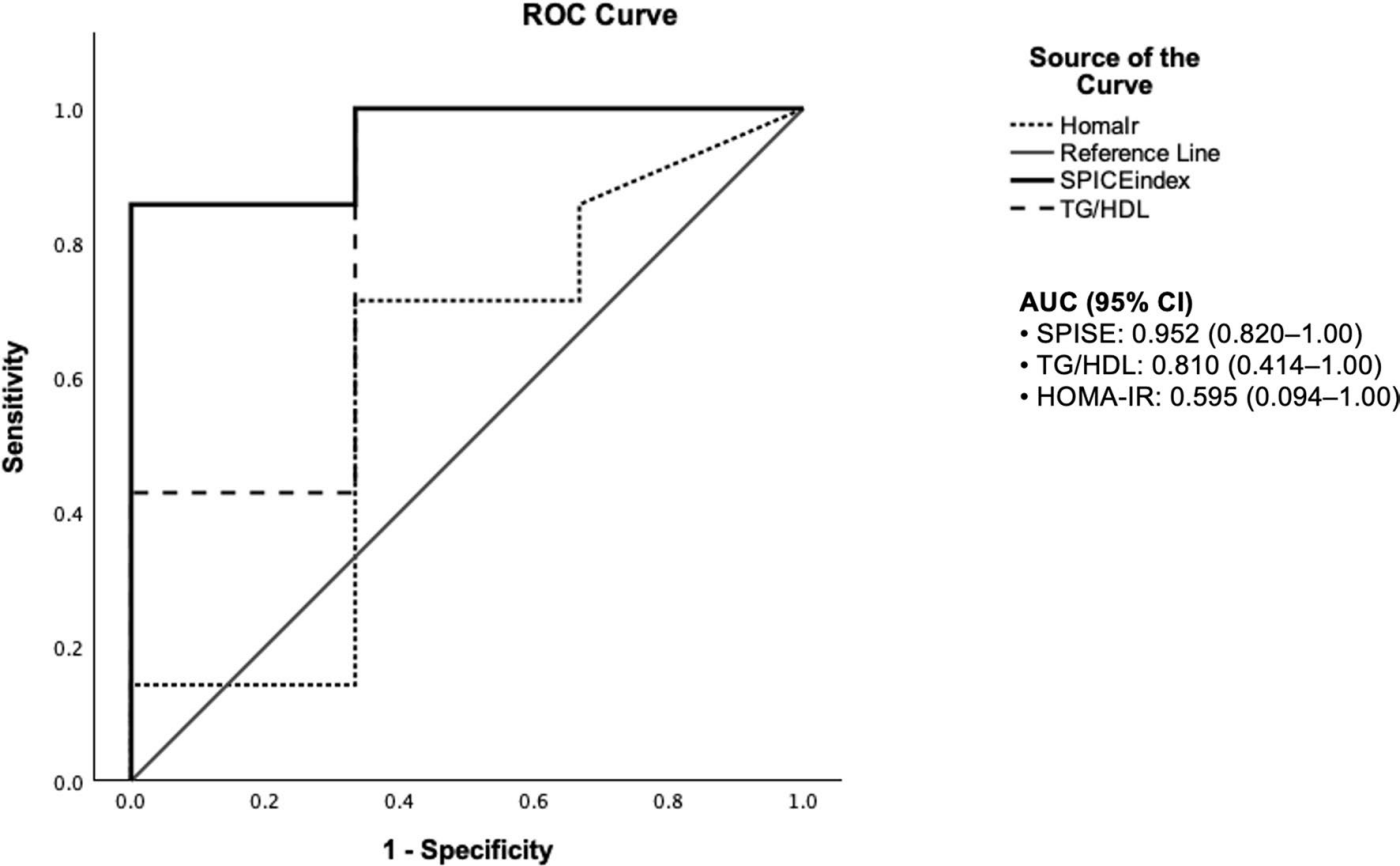
ROC curves for SPISE, TG/HDL, and HOMA-IR to detect metabolic syndrome in BBS patients aged ≥ 10 years. Legend: Pairwise DeLong comparisons of AUCs were not statistically significant: SPISE vs. TG/HDL *p* = 0.532; SPISE vs. HOMA-IR *p* = 0.108; TG/HDL vs. HOMA-IR *p* = 0.545. Youden-optimal cut-offs (sensitivity/specificity) were SPISE ≤ 3.34 (0.86/1.00), TG/HDL ≥ 5.54 (1.00/0.67), and HOMA-IR ≥ 3.59 (0.71/0.67). Detailed operating characteristics (LR+, LR−, PPV, NPV; prevalence 70%) are reported in Supplementary Table S2. *Positive state = metabolic syndrome (1). Given the small sample*,* confidence intervals are wide*

In ROC analyses restricted to adolescents (*n* = 10), SPISE had the highest AUC for metabolic syndrome, exceeding TG/HDL and HOMA-IR (Fig 4). Pairwise DeLong tests were not significant, and the intervals were wide. That result is expected given two design features: the small ROC set limits power for between-curve comparisons, and there is partial overlap between predictor content and the MetS definition (triglycerides, HDL, fasting glucose). Such overlap may inflate AUC values and mask apparent differences between indices. Taken together, these limitations highlight the need for cautious interpretation and external validation in larger, multicenter cohorts [29].

Findings were consistent across analyses. Children with MetS had markedly lower SPISE values than those without, showing clear cross-sectional separation. In covariate-adjusted linear models, SPISE explained a meaningful portion of the variance in MetS z-scores, supporting its use as a marker of continuous burden rather than only a classifier. By contrast, the “perfect” accuracy observed in sensitivity logistic models likely reflects overfitting in a very small sample and should be interpreted with caution.

Placing SPISE alongside insulin-based surrogates is relevant for clinical use. SPISE did not correlate with HOMA-IR but tracked overall MetS burden and a Matsuda-based reference more closely. Similar dissociations have been reported in pediatrics and likely reflect different constructs: HOMA-IR primarily indexes fasting, hepatic insulin resistance and is strongly affected by pubertal physiology, whereas lipid-anchored indices capture downstream dyslipidemia and adiposity [30]. Given the common TG-high/HDL-low pattern in BBS, a lipid-based index is expected to capture a complementary facet of risk rather than the same signal as fasting insulin surrogates. Population-based pediatric studies likewise rank SPISE among the stronger indices for classifying metabolic syndrome across definitions, with associations that persist after adjustment for age, sex, and adiposity [31]. The lack of correlation between SPISE and any insulin-derived index (HOMA-IR, Matsuda, QUICKI; all *p* > 0.30) raises a question about what SPISE is actually measuring in this population. Its correlation with TG/HDL metrics is unsurprising given the shared variables; TG and HDL appear in both, so this cannot be read as evidence of insulin sensitivity estimation. The disconnect from insulin indices may, however, reflect something specific to BBS pathophysiology: dyslipidemia here can arise through ciliary dysfunction independently of insulin resistance [27], so the two types of indices may simply be tracking different processes. Under those conditions, SPISE functions more as a read-out of lipid-driven metabolic deterioration than as a measure of insulin action, still useful for identifying high-risk patients, but not interchangeable with insulin sensitivity testing. Clamp studies would be needed to determine whether that distinction holds across different BBS subtypes and ages.

Using a continuous MetS severity score improved sensitivity and preserved information that binary cut-points would lose. Large datasets show robust cross-sectional and prospective discrimination for type 2 diabetes using age- and sex-weighted MetS-z metrics, supporting continuous scoring for early detection and for longitudinal follow-up [32]. Disease-specific data point the same way in BBS: in a 52-week setmelanotide analysis, mean MetS-Z-BMI improved in parallel with weight-based responses, suggesting that continuous severity scores can register clinically meaningful change in this population [28]. Together with our findings, this supports a pragmatic strategy that pairs a continuous severity score for global burden and trajectory with a simple lipid-based adjunct when insulin testing or OGTT is not feasible. While these findings require multicenter prospective validation and there is acknowledged overlap with lipid components, SPISE may represent a practical adjunct for follow-up, particularly when insulin assays are not feasible. Furthermore, a recent real-world study in individuals with BBS reported substantial improvement in hepatic steatosis (showing 85% resolution or stabilization to grade 1) and increased eGFR after six months of setmelanotide treatment, with hepatic improvements appearing to occur independently of BMI reduction [33].

Reporting Youden-optimal cut points (e.g., SPISE ≤ 3.34 in this cohort) improves transparency and facilitates replication, but clinical adoption should be cautious. Predictive values depend on prevalence, and thresholds may shift with age, pubertal stage, genotype mix, and laboratory methods. Multicenter calibration is required before routine use.

This study has clear strengths: genetic confirmation, standardized phenotyping, and a direct comparison of a clinic-friendly index (SPISE) with insulin-based surrogates and a continuous MetS severity score. However, the single-center, retrospective design and the small sample (ROC subsets = 10) widen confidence intervals and may overstate separation. Pubertal heterogeneity and concurrent therapies could also confound associations. Finally, the study was underpowered to assess genotype–phenotype differences in metabolic risk, which have been suggested and merit systematic evaluation [4, 31]. Additionally, partial overlap between predictors and the outcome definition (lipids within both SPISE and MetS) may inflate discrimination and should be kept in mind when comparing indices. An important interpretive limitation specific to BBS is the potential contribution of ciliopathy-driven organ damage to cardiometabolic parameters, independent of insulin sensitivity. Renal anomalies were present in 10/14 participants, and chronic kidney disease requiring renal transplantation was documented in two patients. Renal impairment can independently elevate triglycerides, reduce HDL-cholesterol, and raise blood pressure through non-insulin-mediated pathways, thereby inflating SPISE-based and lipid-derived MetS metrics. Similarly, hepatic steatosis was present in 7/14 patients, a finding that may partly reflect primary ciliary dysfunction of hepatocytes, as recently suggested [33], rather than exclusively adiposity-driven insulin resistance. The small sample size precluded statistical adjustment for these confounders, and we cannot determine what proportion of the observed associations between SPISE and cardiometabolic burden is attributable to ciliopathy-specific mechanisms versus adiposity-driven insulin resistance. This represents a fundamental limitation of the current design and underscores the necessity of organ-function-stratified analyses in future, larger cohorts.

## Conclusions

Children with Bardet–Biedl syndrome show a high cardiometabolic burden from early childhood. In this cohort, the MetS z-score captured clustered risk well and was useful for follow-up. SPISE was aligned with lipid markers and, in adolescents, distinguished metabolic syndrome better than TG/HDL and HOMA-IR. The MetS z-score can be used for overall risk tracking, with SPISE as a low-cost adjunct when insulin assays or an OGTT are not available. Larger multicentre studies with standardised pubertal staging, longitudinal outcomes, and genotype-stratified analyses are needed to define BBS-specific thresholds.

## Supplementary Information

Below is the link to the electronic supplementary material.

Supplementary Material 1

Supplementary Material 2

## Data Availability

Data supporting the findings are available from the corresponding author upon reasonable request.

## Abbreviations

ACMG: American College of Medical Genetics and Genomics
AUC: Area under the curve
BBS: Bardet–Biedl syndrome
BMI: Body mass index
BMI SDS: Body mass index standard deviation score
BP: Blood pressure
CI: Confidence interval
eGFR: Estimated glomerular filtration rate
HbA1c: Hemoglobin A1c
HDL-C: High-density lipoprotein cholesterol
HOMA-B: Homeostatic model assessment of β-cell function
HOMA-IR: Homeostatic model assessment of insulin resistance
HT: Hypertension
I/G: Insulin-to-glucose ratio
IQR: Interquartile range
IRI: Insulin-resistance index
ISI: Insulin sensitivity index (OGTT-derived)
LDL-C: Low-density lipoprotein cholesterol
MetS: Metabolic syndrome
MetS z-score: Metabolic syndrome severity z-score
NGS: Next-generation sequencing
OGTT: Oral glucose tolerance test
QUICKI: Quantitative insulin sensitivity check index
ROAD: Rare Obesity Advanced Diagnosis (genetic support program)
ROC: Receiver operating characteristic
SD: Standard deviation
SPISE: Single-Point Insulin Sensitivity Estimator
TG: Triglycerides
TG/HDL: Triglyceride-to-HDL ratio
