# Supplementary material for: Assessment of cardiometabolic risk using single point insulin sensitivity estimator (SPISE) in pediatric Bardet–Biedl Syndrome: a pilot study
Source: Orphanet J Rare Dis. 2026 Aug 6;21:266. doi: 10.1186/s13023-026-04353-y (PMC13445732; doi:10.1186/s13023-026-04353-y)
Supplement: Supplementary file 1 [file 13023_2026_4353_MOESM1_ESM.docx]

**Supplementary Table S1.** Detailed clinical, anthropometric, hormonal, and metabolic characteristics of 14 genetically confirmed Bardet-Biedl syndrome patients.

| **Case** | **1** | **2** | **3** | **4** | **5** | **6** | **7A*** | **7B*** | **8** | **9** | **10A*** | **10B*** | **11** | **12** |  |
| --- | --- | --- | --- | --- | --- | --- | --- | --- | --- | --- | --- | --- | --- | --- | --- |
| ***AT ADMISSION*** | | | | | | | | | | | | | | | |
| **Age(years)** | 2.9 | 4.9 | 2.5 | 6.6 | 11.3 | 7 | 13.7 | 18.6 | 3.7 | 5 | 0.7 | 1.6 | 5.9 | 0.5 |  |
| **Sex** | M | F | F | F | M | M | M | M | M | F | M | F | F | F |  |
| **Consanguinity** | Yes | Yes | No | Yes | Yes | Yes | No | No | Yes | Yes | Yes | Yes | Yes | Yes |  |
| **Height SDS** | -1.2 | 0.4 | 0.02 | -4.7 | 0.9 | -1.5 | 0.8 | 1.4 | - | -0.4 | 0.7 | 0.2 | -2.5 | -1.1 |  |
| **BMI (kg/m^2^)/SDS/**  **BMI %** | 24.7/3.9/ 132 | 29.2/4.27/159 | 30.7/5.7/167 | 23.9/2.7/122 | 32/2.7/128 | 22.6/2.7/118 | 27.9/1.9/104 | 36.4/2.7/123 | - | 21.7/2.7/117 | 24.8/3.8/121 | 21.2/2.6/110 | 20.1/2/105 | 35.6/7/180 |  |
| **BBS subtype** | BBS1 | BBS2 | BBS2 | BBS2 | BBS2 | BBS4 | BBS5 | BBS5 | BBS7 | BBS8 | BBS9 | BBS9 | BBS9 | BBS10 |  |
| **Genetic analysis result ****  **(Homozygous)** | c.48-1G>A p.? | c.1770_1791delins15 p.(Phe590Leufs*29) | c.940del p.(Ile314Serfs*11) rs587777824 | c.1864C>T p.(Arg622*) | c.940delA p.(Ile314Serfs*11) | c.332+2_332 +3insTT | c.974A>G p.(E325G) | c.974A>G p.(E325G) | c.849+1G>T | c.661A>G p.(Asn221Asp) | c.310del p.(Cys104Valfs*20) | c.310del p.(Cys104Valfs*20) | c.310del p.(Cys104Valfs*20) | c.661A>G p.(Asn221Asp) |  |
| ***AT LAST VISIT*** | | | | | | | | | | | | | | | |
| **Age (years)** | 14.5 | 11.5 | 6.9 | 11.9 | 16.5 | 7.5 | 15.4 | 18.6 | 15.8 | 14.9 | 7.5 | 1.6 | 11 | 10.2 |  |
| **Height SDS** | 0.44 | -1.4 | 1.4 | -5 | 1 | -1.5 | 0.2 | 1.4 | -2 | 0.8 | -0.6 | 0.2 | -1.6 | 1.6 |  |
| **BMI (kg/m^2^)/SDS/**  **BMI %** | 34.8/2.8/127 | 33.2/3.1/136 | 35.4/3.7/180 | 35.8/3.4/145 | 33.4/2.4/116 | 23.6/2.8/122 | 30.5/2.1/110 | 36.4/2.7/123 | 37/3/132 | 38.8/3.9/150 | 29.2/3.5/150 | 21.2/2.6/110 | 25.9/2/110 | 36.3/3.5/158 |  |
| **WC SDS** | 4.1 | NA | 4.5 | 4 | 3.8 | 3.7 | 2.8 | 4.5 | 4.4 | 4.5 | 3.8 | 3 | 3 | 4.9 |  |
| **Tanner stage/**  **Pubertal onset age (years)** | T5/9.1 | T3/8 | T1 | T2 /11.9 | T5/11.8 | T1 | T5 | T5 | T5 | T5/8.8  Menarche started at age 15 | T1 | T1 | T3 | T3 |  |
| **TG mg/dl** | 263 | 800 | 210 | 352 | 347 | 77 | 233 | 183 | 79 | 137 | 223 | 91 | 418 | 288 |  |
| **HDL mg/dl** | 36 | 39 | 35 | 25 | 27 | 34 | 27 | 31 | 46 | 43 | 30 | 34 | 42 | 38 |  |
| **HOMA-IR** | 10.5 | 49.2 | 2.5 | 2.1 | 3.9 | 2.8 | 4.4 | 2.1 | 2.1 | 13 | 2 | 3.6 | 3.3 | 7.8 |  |
| **OGTT result** | IR | T2DM | NA | IR | IR | IR | IR | NA | IR | IR | IR | NA | IR | IR |  |
| **LH/FSH mIU/L** | 3.9/4.6 | 3.7/0.36 | 1.1/<1 | 5.5/6 | 6.2/7.4 | <1/<1 | 4.2/5.8 | 2.9/4.8 | 5.1/5.5 | 5.7/9 | 1.2/0.3 | 3.9/0.3 | 7.4/1.4 | 6.9/7.2 |  |
| **E2 pg/ml /T ng/ml** | 1.2 | 13 | <5 | 18 | 3.7 | <0.12 | 3.2 | 2.7 | 3 | 33 | 0.03 | <5 | 5 | 5 |  |
| **Thyroid disease** | No | Central hypothyroidism | Subclinical hypothyroidism | No | No | Subclinical hypothyroidism | No | No | Central hypothyroidism | No | No | No | Hashimoto disease | Primary hypothyroidism |  |
| **MetS /**  **MetS risk score** | Yes /1.9 | Yes /1.6 | NA^***^ /1.6 | Yes /1.6 | Yes /1.6 | NA^***^ /0.4 | Yes /1.7 | Yes /1.7 | No /0.7 | No /1 | NA^***^ /1.9 | NA^***^/0.3 | No /1 | Yes /1.9 |  |
| **Hepatosteatosis** | G2-3 | G2 | No | No | G2-3 | - | No | G2 | - | G3 | - | No | G2 | G2 |  |

Abbreviations: SDS: standard deviation score; BMI: body mass index; BMI %: The ratio of BMI to the 95th percentile value; WC: waist circumference; F: Female; G: Grade of hepatosteatosis; TG: triglycerides; HDL: high-density lipoprotein; IR: insulin resistance; N: normal; NA: non available data; M: Male; T2DM: type 2 diabetes mellitus; OGTT: oral glucose tolerance test; E2: estradiol; T: total testosterone; MetS: Metabolic syndrome)

*Cases from same family ** In cases 2, 10, and 12, a heterozygous PCSK1 variant p.(Asn221Asp) was also identified ^***^Not applicable, because age is <10 years
