# Supplementary material for: Assessment of cardiometabolic risk using single point insulin sensitivity estimator (SPISE) in pediatric Bardet–Biedl Syndrome: a pilot study
Source: Orphanet J Rare Dis. 2026 Aug 6;21:266. doi: 10.1186/s13023-026-04353-y (PMC13445732; doi:10.1186/s13023-026-04353-y)
Supplement: Supplementary file 2 [file 13023_2026_4353_MOESM2_ESM.docx]

**Supplementary Table S2.** Diagnostic operating characteristics at Youden-optimal cut-offs (prevalence assumed 70%)

| Index (Cut-Off) | Sensitivity | Specificity | LR+ | LR− | PPV (70%) | NPV (70%) |
| --- | --- | --- | --- | --- | --- | --- |
| **SPISE ≤ 3.34** | 0.86 | 1.00 | ∞ | 0.14 | 1.00 | 0.75 |
| **TG/HDL ≥ 5.54** | 1.00 | 0.67 | 3.03 | 0.00 | 0.88 | 1.00 |
| **HOMA-IR ≥ 3.59** | 0.71 | 0.67 | 2.15 | 0.43 | 0.83 | 0.50 |

**Notes.**

- LR+ = sensitivity / (1 − specificity); LR− = (1 − sensitivity) / specificity.
- PPV = (Se×Prev) / (Se×Prev + (1−Sp)×(1−Prev)); NPV = (Sp×(1−Prev)) / ((1−Se)×Prev + Sp×(1−Prev)), with Prev = 0.70.
- “∞” arises when **specificity = 1.00** (no false positives in this sample). With very small samples, some journals prefer reporting a very large number (e.g., “>10”) and noting the zero cell.
- These values are point estimates; CIs are wide due to small n.
